# Chromophore-loaded CRALBP mutant proteins restore rod function in chromophore-deficient mice

**DOI:** 10.1016/j.omta.2026.201701

**Published:** 2026-02-17

**Authors:** Alexander V. Kolesnikov, Walter Aeschimann, John Cullity, Maximilian Halabi, Philip D. Kiser, Achim Stocker, Vladimir J. Kefalov

**Affiliations:** 1 Gavin Herbert Eye Institute – Brunson Center for Translational Vision Research, Department of Ophthalmology and Visual Sciences, Irvine, CA 92697, USA; 2 Department of Chemistry, Biochemistry and Pharmaceutical Sciences, Freiestrasse 3, 3012 Bern, Switzerland; 3 NanoRetinal Inc., 41 University Drive, Suite 400, Newtown, PA 18940, USA; 4 Department of Physiology and Biophysics, University of California, Irvine, Irvine, CA 92697, USA; 5 Research Service, VA Long Beach Medical Center, Long Beach, CA 90822, USA

**Keywords:** visual chromophore, nanoparticles, opsin, pigment regeneration, rod photoreceptors, CRALBP

## Abstract

Visual function depends critically on the supply of visual chromophore, 11-*cis*-retinal, to the photoreceptor cells in the retina. Chromophore deficiency due to aging or mutations affecting its delivery and recycling in the eye by the retinal pigment epithelium (RPE) and the Müller cells cause a wide range of visual disorders. The cellular retinaldehyde-binding protein (CRALBP), expressed in both compartments, crucially aids in accelerating the recycling of visual chromophore. Here, we explored the potential of chromophore-loaded CRALBP in restoring vision in chromophore-deficient mice. We tested wild-type human CRALBP and its redox-sensitive A212C:T250C mutant, both pre-loaded with 9-*cis*-retinal, for their efficacy in delivering chromophore to the retina and restoring rod photoreceptor function in RPE65-deficient mice that cannot produce visual chromophore. We observed robust restoration of rod function both in the isolated retina treated with chromophore-loaded CRALBP and *in vivo* after a single intravitreal injection of wild-type or mutant CRALBP proteins. Notably, the recovery of rod visual function after exposure to bright light that photoactivated most of the visual pigment was greatly accelerated in CRALBP-treated RPE65-knockout mice compared to wild-type control mice. Together, our results highlight the therapeutic potential of CRALBP complexes in efficiently delivering visual chromophore to retinal rod photoreceptors.

## Graphical abstract

**Figure:**
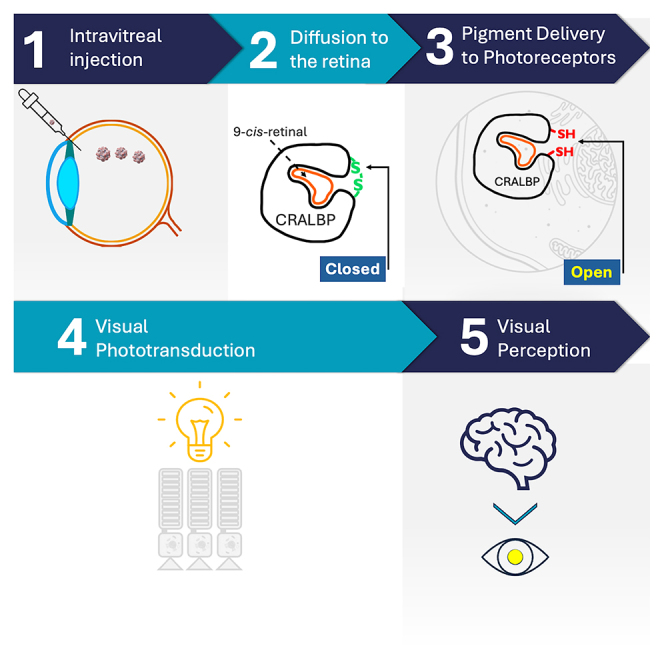

## Teaser

Kolesnikov and colleagues demonstrate that visual chromophore-loaded cellular retinaldehyde-binding protein complexes restore rod photoreceptor function and dark adaptation in chromophore-deficient mice. This protein-based delivery approach offers a potential therapeutic strategy for treating various visual disorders caused by impaired chromophore recycling in the retina and adjacent retinal pigmented epithelium cells.

## Introduction

The indispensable role of vitamin A aldehyde, specifically 11-*cis*-retinal, as the primary chromophore in mammalian vision is well established. Upon combining with opsin receptors, it forms rhodopsin, the light-sensitive visual pigment crucial for rod photoreceptor cell function. This molecular complex initiates vision through photon absorption, leading to chromophore isomerization into all-*trans*-retinal and activation of the phototransduction cascade.^1^ Subsequent investigations identified cellular retinaldehyde-binding protein (CRALBP) in the soluble fraction of bovine retina and retinal pigment epithelium (RPE). CRALBP was shown to exhibit maximal binding affinity for 11-*cis*-retinal among *cis*-*trans* isomers, hinting at its potential involvement in chromophore recycling.^2^

Further research explored the formation of various retinal-protein complexes, including rhodopsin (11-*cis*-retinal), isorhodopsin (9-*cis*-retinal), and isorhodopsin II (9,13-di-*cis*-retinal), shedding light on the molecular intricacies of visual pigment assembly.^3^ These discoveries laid the groundwork for exploring CRALBP’s role in the regeneration of bleached rhodopsin, as proposed by subsequent investigations.^3^^,^^4^^,^^5^

Isolation and characterization of CRALBP from bovine retina revealed its high specificity for geometric isomers of retinal, particularly 11-*cis*-retinal and 9-*cis*-retinal, crucial for the visual cycle.^6^^,^^7^ The subsequent cloning and sequencing of bovine and human CRALBP cDNAs provided deeper insights into its genetic basis and paved the way for associating CRALBP mutations with various retinal diseases.^8^^,^^9^^,^^10^^,^^11^^,^^12^

Structural studies further provided critical insights into CRALBP’s conformational dynamics and its interaction with various retinoids, enhancing our understanding of its functional mechanisms.^13^^,^^14^^,^^15^ Furthermore, its pivotal role in the retinoid (visual) cycle, orchestrating the recycling of visual chromophore from all-*trans*-retinal to 11-*cis*-retinal, was elucidated in subsequent biochemical and *in vivo* experiments.^12^^,^^16^^,^^17^^,^^18^

Recent investigations employing knockout mouse models have underscored the importance of CRALBP in maintaining rod- and cone-driven vision and particularly in accelerating dark adaptation of these photoreceptors, further solidifying its significance in retinal physiology.^19^^,^^20^^,^^21^^,^^22^ Here, we present the engineering and production of a mutant CRALBP protein harboring a dual replacement of alanine 212 and threonine 250 within the wild-type structure of human CRALBP by cysteine residues (A212C:T250C).^13^ The 212C:250C pair of cysteines is able to reversibly form a disulfide bond subsequent to sequestering 9-*cis-*retinal within its ligand-binding pocket. In the oxidized state, the ligand complex exhibits significantly increased *in vitro* photostability to daylight with similar biophysical properties compared to its wild-type counterpart under reducing conditions.^13^ Our objective is to employ the oxidized state of the complex as an *in vivo* storage platform for the safe and efficient delivery of 9-*cis*-retinal to retinal photoreceptors.

## Results

### Design of a CRALBP variant with a redox-sensitive switch

**Figure 1:**
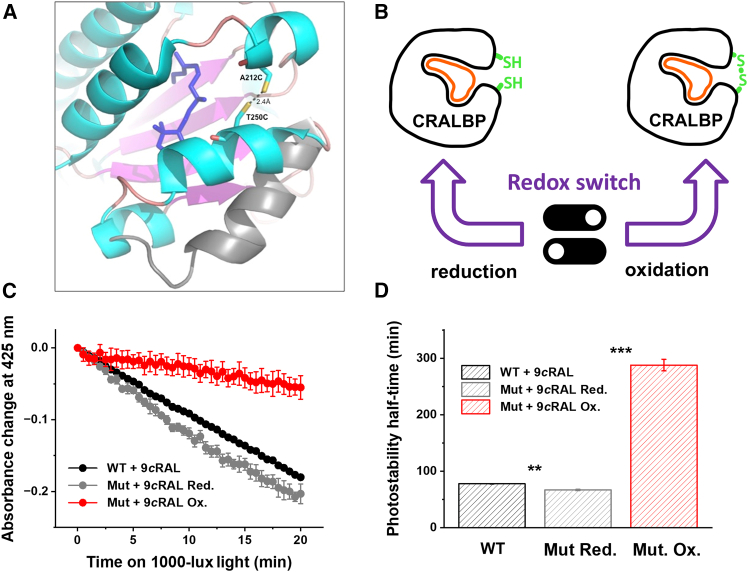
Design and analysis of the mutant A212C:T250C CRALBP (A) Close-up view of the mobile gate region of the molecular model of native CRALBP in its “closed” conformation (3HY5.pdb)^13^ showcases the di-cysteine A212C:T250C mutation’s position and orientation, depicted as sticks. Mutations were introduced using Coot, and side-chain rotamers were organized using the program’s rotamer library. Distances ranging from 1.9 to 3.1 Å between the terminal sulfhydryl groups of C212 and C250 suggest favorable conditions for disulfide bond formation (2.4 Å) under oxidizing conditions. A 3-dimensional overlay with the X-ray structural model of the mobile gate moiety in the “open” state (gray helix) of the apo-form of alpha-TTP (1oiz.pdb; closest structural homologue of CRALBP) vividly demonstrates the magnitude of the conformational change from the “closed” to the “open” state. The figure was adapted from Stocker.^23^ (B) Engineered di-cysteine A212C:T250C mutant of CRALBP loaded with 9-*cis*-retinal in its oxidized state features a redox switch that reversibly locks the visual pigment, thereby substantially increasing its half-life compared to its reduced state and to native CRALBP. (C) Photostability of wild-type CRALBP (*n* = 3) and both reduced (*n* = 3) and oxidized states of the di-cysteine CRALBP mutant in complex with 9-*cis*-retinal measured during exposure of the samples to a 1,000-lux light. Values are means ± SEM. Error bars for some points are smaller than the symbol size. The figure was adapted from Stocker.^23^ (D) Photostability half-life times (t_1/2_) of CRALBP/retinal complexes calculated from the data in (C) using the standard formula for first-order reactions: (t_1/2_) = ln(2)/R. The figure was adapted from Stocker.^23^ Values are means ± SEM (*n* = 3 for all groups, ∗∗*p* < 0.01, ∗∗∗*p* < 0.001).

We engineered a novel CRALBP variant containing a redox-sensitive switch, achieved by the dual replacement of alanine 212 and threonine 250 within the native human CRALBP structure with cysteine residues (A212C:T250C). Both residues are solvent accessible with their side chains being sufficiently similar to the one of cysteine and thus were expected to minimize adverse effects after introduction through site-directed mutagenesis.^23^ The A212C substitution is located in helix 10 of the protein’s core, while the T250C substitution is in the helix 12 within the mobile gate moiety spanning residues E243–E254, as illustrated in Figure 1A.

Dissociation constants of wild-type CRALBP for 11-*cis*-retinal/retinol (Kd ≈ 21 nM/53 nM) and for 9-*cis*-retinal (Kd ≈ 51 nM) have been reported.^6^^,^^7^ Upon ligand binding, the mobile gate moiety of CRALBP undergoes a conformational change, adopting a “closed” state bringing helices 12 and 10 into a close proximity via van der Waals interactions as observed in the CRALBP crystal structure. In this more compact conformation, the mutated residues C212 and C250 of the A212C:T250C protein are ideally positioned to form a covalent disulfide bond, with their side-chain sulfur atoms opposing each other at a distance of 2.4 Å within the helical region termed mobile gate interface. This arrangement facilitates the reversible formation of a disulfide linkage,on one hand, upon exposure to atmospheric oxygen or other suitable oxidizing agents through the loss of two electrons. On the other hand, it allows for the subsequent re-reduction of the S-S bond by suitable reducing agents or the reducing environment within a cell (Figure 1B).

### Production and biophysical characterization of CRALBP proteins

Native CRALBP and the di-cysteine A212C:T250C mutant of CRALBP were overexpressed in *E. coli* BL21(DE3). After affinity purification on Ni-NTA, both apo-proteins were loaded with 9-*cis*-retinal and subjected to preparative gel permeation chromatography (GPC). Upon introduction of 9-*cis*-retinal to native CRALBP, four distinct peaks emerged, delineating the ligand complexes of the monomeric protein and its associated high-molecular-weight (HMW) aggregates (Figure S1). Further analysis of the preparative GPC revealed analogous chromatographic profiles with similar elution volumes when loading the apo di-cysteine A212C:T250C mutant of CRALBP with 9-*cis*-retinal. Subsequent re-chromatography of the collected peak fractions was conducted by analytical GPC (Figure S2).

The constituents of the analytical GPC peak fractions were further elucidated through dynamic light scattering (DLS), revealing the presence of three discernible populations of oligomeric CRALBP aggregates: those with a substantial (super) HMW (SHMW) with an apparent diameter of 28.3 ± 2.1 nm, those classified as HMW with an apparent diameter of 13.5 ± 0.5 nm, and dimers with an apparent diameter of 8.7 ± 2.4 nm, alongside the monomeric CRALBP with an apparent diameter of 6.5 ± 1.5 nm (Figure S3).

The DLS analysis also highlighted that the HMW A212C:T250C complex exhibits the narrowest size distribution among the four peak fractions. However, its analytical GPC trace unveiled the presence of notable quantities of monomeric A212C:T250C complex (Figure S2), suggesting reversible equilibration between the monomeric and HMW states. In addition, the fraction of the dimeric A212C:T250C complex lacked an adequate concentration of 9-*cis*-retinal. Consequently, further investigation into the complexes of HMW and of dimer was not pursued.

To assess the extent of ligand loading, we compared ultraviolet-visible (UV-vis) absorption spectra between monomeric native CRALBP and the monomeric A212C:T250C mutant, both bound to 9-*cis*-retinal (Figure S4). By normalizing the absorption spectra to the protein’s maximum absorption around 280 nm, we could analyze the absorption peak at 400 nm, indicative of bound 9-*cis*-retinal. The optimal spectral ratio of ε280/ε400 for fully saturated CRALBP in complex with 9-*cis*-retinal was earlier found to be 2.2.^24^ In our experiments, we observed ratios of 2.3 for native CRALBP and 2.2 for the A212C:T250C mutant, suggesting the formation of fully saturated 1:1 complexes.

### Modulation of photostability of A212C:T250C mutant CRALBP protein

To investigate the functionality of the two cysteine residues in the A212C:T250C mutant protein, we assessed the photostability of the monomeric mutant protein in a photo-isomerization assay developed previously.^7^ A212C:T250C protein was loaded with 9-*cis*-retinal under reducing conditions. Subsequently, the complex was oxidized by performing GPC in the presence of 5 mM oxidized glutathione (GSSG).

The photostability of wild-type CRALBP and of the A212C:T250C mutant both in complex with 9-*cis*-reinal was monitored at room temperature under moderate light intensity (1,000 lux). The samples were exposed to a 100-W daylight bulb in a darkroom setting. UV-vis absorption spectra were recorded at half-minute intervals over a total duration of 20 min using a diode array UV-vis spectrophotometer (Figure 1C).

A comparison of reducing and oxidizing conditions revealed a substantial enhancement in photostability of 9-*cis*-retinal when bound to the oxidized state of the A212C:T250C mutant compared with the wild-type complex (Figure 1D). We observed a ∼4.3-fold increase in the photostability half-life between the oxidized and reduced forms of the 9-*cis*-retinal-laden A212C:T250C mutant. Our findings underscore the proficiency of the A212C:T250C mutant protein in modulating photostability through oxidation and reduction processes. Under oxidizing conditions, the *cis*-retinoid is trapped within the ligand-binding cavity of the protein, as the mobile gate is locked in its “closed” position. The tightly bound *cis*-retinal may still absorb photons, but its transition from the bent *cis*-conformation to the elongated *trans*-conformation is sterically hindered (Figure 1B).

These results indicate that the oxidized state of A212C:T250C mutant provides an amplified photostability for 9-*cis*-retinal in an oxidizing environment, like during transit through extracellular compartments. Additionally, A212C:T250C protein is anticipated to substantially hinder the premature release of *cis*-retinal from its binding pocket under these circumstances, surpassing the performance of its wild-type form in efficiently delivering chromophore to photoreceptors.

### Delivery of CRALBP proteins to isolated mouse retinas

To evaluate the ability of recombinant CRALBP proteins to deliver chromophore to photoreceptors, we first treated chromophore-deficient isolated retinas of *Rpe65*^−/−^ mice with monomeric and SHMW fractions of native CRALBP and A212C:T250C mutant variant loaded with 9-*cis*-retinal. We then analyzed rod photoreceptor function with transretinal (*ex vivo*) electroretinography (ERG) recordings. The recordings were made in the presence of postsynaptic inhibitors to block the contribution of ON bipolar cell-driven ERG b-wave, thus allowing us to isolate the rod photoresponse component.^25^ Cone photoreceptors are largely degenerated in this mouse model and produce negligible light responses at the age of 1 month and later.^26^^,^^27^

**Figure 2:**
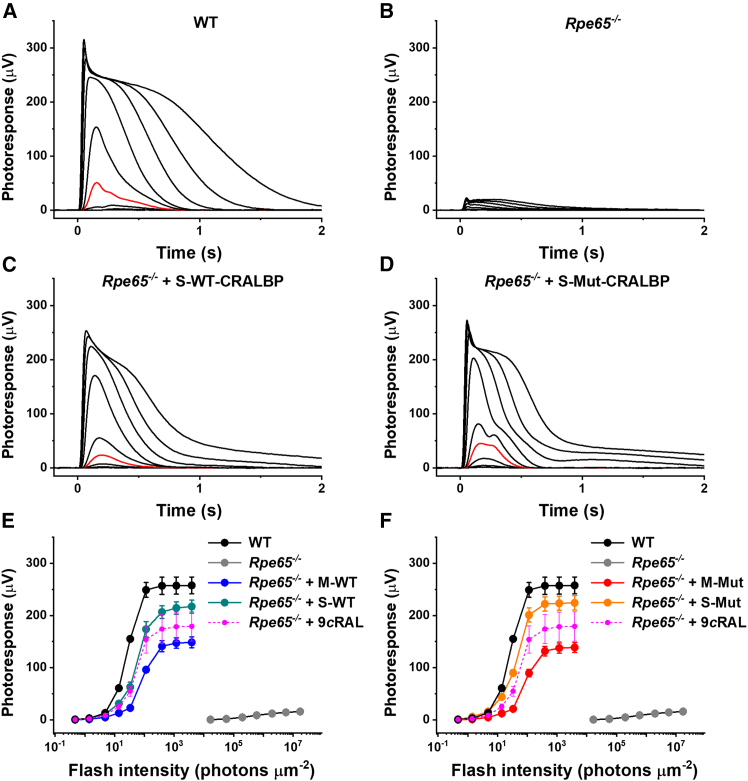
Treatment of isolated retinas with CRALBP proteins loaded with 9-*cis*-retinal restores sensitivity of chromophore-deficient mouse rods (A) Representative family of rod *ex vivo* ERG responses from wild-type (C57BL/6J) mouse retina. Test flashes of 505-nm light with intensities of 0.5, 1.4, 4.8, 14 (red trace), 33, 114, 392, 1.2 × 10^3^, and 3.9 × 10^3^ photons μm^−2^ were delivered at time 0. (B) Representative family of rod *ex vivo* ERG responses from untreated *Rpe65*^−/−^ mouse retina. Test flashes of 505-nm light with intensities of 6.9 × 10^3^, 1.7 × 10^4^, 5.7 × 10^4^, 2.0 × 10^5^, 6.0 × 10^5^, 2.0 × 10^6^, and 5.7 × 10^6^ photons μm^−2^ were delivered at time 0. (C) Representative family of rod *ex vivo* ERG responses from retinas of *Rpe65*^−/−^ animals treated with SHMW wild-type CRALBP + 9-*cis*-retinal. Test flashes of 505-nm light with intensities of 0.5, 1.4, 4.8, 14 (red trace), 33, 114, 392, 1.2 × 10^3^, and 3.9 × 10^3^ photons μm^−2^ were delivered at time 0. (D) Representative family of rod *ex vivo* ERG responses from retinas of *Rpe65*^−/−^ animals treated with SHMW A212C:T250C mutant CRALBP + 9-*cis*-retinal. Test flashes of 505 nm light with intensities of 0.5, 1.4, 4.8, 14 (red trace), 33, 114, 392, 1.2 × 10^3^, and 3.9 × 10^3^ photons μm^−2^ were delivered at time 0. (E) Averaged rod intensity-response functions (mean ± SEM) for wild-type control (*n* = 4), untreated *Rpe65*^−/−^ (*n* = 6), *Rpe65*^−/−^ treated with monomeric wild-type CRALBP (M-WT) + 9-*cis*-retinal (*n* = 4), and SHMW wild-type CRALBP (S-WT) + 9-*cis*-retinal-treated *Rpe65*^−/−^ (*n* = 8) retinas. (F) Averaged rod intensity-response functions (mean ± SEM) for wild-type control (*n* = 4, same data as in E), untreated *Rpe65*^−/−^ (*n* = 6, same data as in E), *Rpe65*^−/−^ treated with monomeric A212C:T250C mutant CRALBP (M-Mut) + 9-*cis*-retinal (*n* = 6), and SHMW A212C:T250C mutant CRALBP (S-Mut) + 9-*cis*-retinal-treated *Rpe65*^−/−^ (*n* = 6) retinas. Magenta traces in (E and F) represent the data from retinas treated with 9-*cis*-retinal alone (*n* = 6). Error bars for some points in (E and F) are smaller than the symbol size.

Consistent with previously reported results,^26^^,^^28^ chromophore-deficient rods in *Rpe65*^−/−^ mice exhibited ∼10-fold smaller responses to test flashes of green light and were desensitized by approximately ∼2,000-fold, as compared with those from wild-type (C56BL/6J) animals (Figures 2A, 2B, 2E, and 2F). Importantly, retinas treated with both native and A212C:T250C mutant CRALBP demonstrated robust recovery of their rod responses in these conditions (Figures 2C–2F). Notably, rod recovery for both CRALBP variants was higher after the treatment with SHMW compared to monomeric CRALBP fractions, and it was comparable to that observed in retinas treated with 9-*cis*-retinal alone (magenta traces). Surprisingly, the overall degree of response recovery was similar upon treatment with wild-type and di-cysteine CRALBP mutant samples. Thus, both native and mutant CRALBP proteins could efficiently deliver 9-*cis*-retinal to free opsin in the rods of chromophore-deficient isolated retinas of *Rpe65*^−/−^ mice, reconstitute the pool of their visual pigment, and restore rod photoreceptor function.

### Delivery of CRALBP proteins to mouse retinas *in vivo*

**Figure 3:**
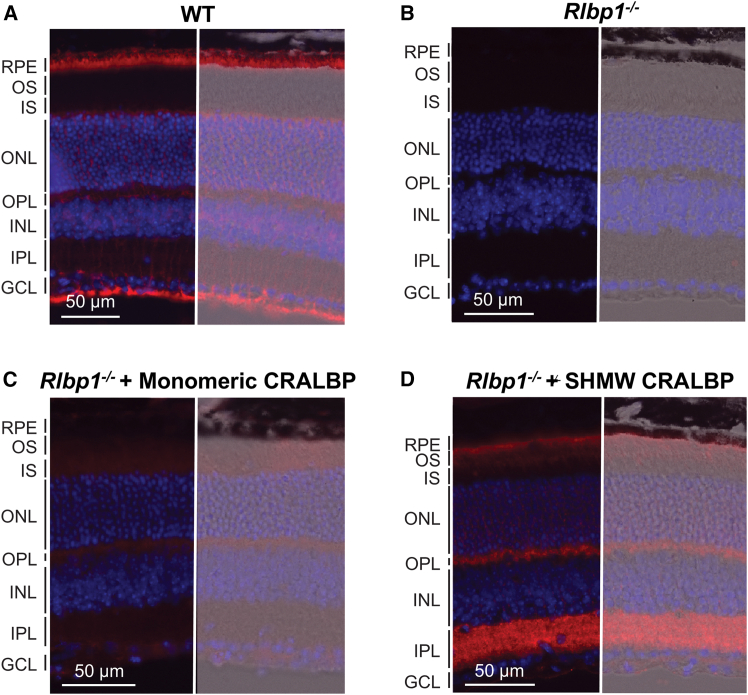
Delivery of A212C:T250C mutant CRALBP proteins to the retina via intravitreal injections (A) Wild-type control, (B) untreated *Rlbp1*^−/−^ mouse, (C) *Rlbp1*^−/−^ mouse treated with monomeric mutant CRALBP (+9-*cis*-retinal), and (D) *Rlbp1*^−/−^ mouse treated with SHMW mutant CRALBP (+9-*cis*-retinal). Confocal images of retinal cross-sections in transmitted light, with bright-field images superimposed on the right. CRALBP expression was examined by IHC with an anti-CRALBP antibody (red), and cell nuclei were stained with DAPI (blue). OS, photoreceptor outer segments; IS, photoreceptor inner segments; ONL, outer nuclear layer; OPL, outer plexiform layer; INL, inner nuclear layer; IPL, inner plexiform layer; GCL, ganglion cell layer.

We next used intravitreal injections to deliver 2 μL chromophore-loaded wild-type or A212C:T250C mutant CRALBP solution at a concentration of 60 μg/μL to the retina of living mice. The efficiency of the delivery of CRALBP to the photoreceptor layer was assessed by immunohistochemistry (IHC) 36–48 h post-injection. As expected, CRALBP was abundantly expressed in the RPE and Muller cells in wild-type control mice (Figure 3A), while it was absent from both cell types in *Rlbp1*^−/−^ animals lacking this protein (Figure 3B). Retinal sections from CRALBP-injected *Rlbp1*^−/−^ mouse eyes demonstrated robust CRALBP signal when the animals were treated with the monomeric form of di-cysteine mutant protein (Figure 3C). The administration of SHMW mutant variant resulted in even greater delivery of CRALBP to photoreceptors in which all compartments, from outer segments to synaptic terminals, were labeled (Figure 3D). Notably, the distribution of injected CRALBP was clearly not uniform and distinct from that of the endogenous RPE- and Muller cells-expressed CRALBP. The difference in staining patterns between the monomeric and SHMW CRALBP forms is consistent with the difference in rod response restoration, which was more robust with the SHMW than monomeric CRALBP in isolated retina (Figure 2). The effective delivery of CRALBP to the retina by intravitreal injection allowed us to further investigate the restoration of rod function with CRALBP proteins under *in vivo* conditions.

**Figure 4:**
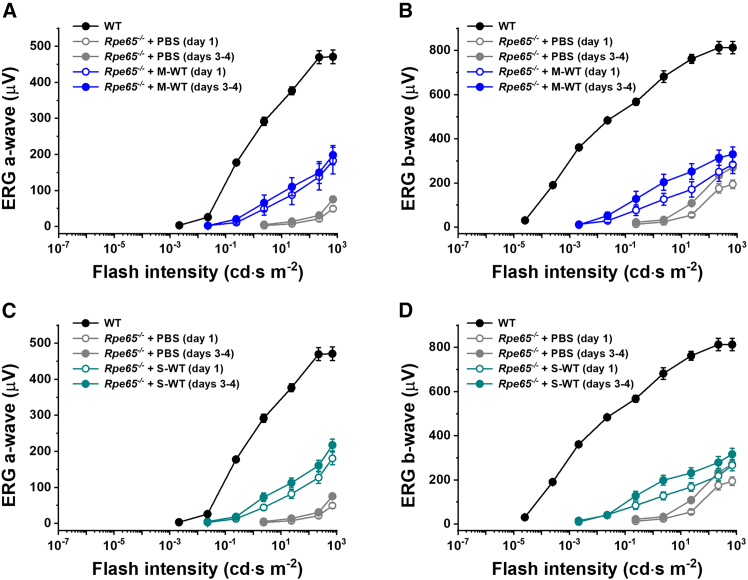
Recovery of scotopic visual function in *Rpe65*^−/−^ mice treated with wild-type CRALBP/9-*cis*-retinal complexes The rod function was studied by *in vivo* ERG. Data show averaged scotopic intensity-response functions (mean ± SEM) for ERG a-wave (A and C) and b-wave (B and D) recorded 1 day (open symbols) and 3–4 days (closed symbols) post-injection in wild-type control (black; untreated, *n* = 8 eyes) and *Rpe65*^−/−^ control (gray; PBS injected, *n* = 9 eyes for post-injection day 1, *n* = 4 eyes for post-injection days 3–4) mice, as well as in *Rpe65*^−/−^ animals injected with wild-type monomeric CRALBP (A and B) (blue symbols, *n* = 12 eyes for post-injection day 1, *n* = 10 eyes for post-injection days 3–4) or with wild-type SHMW CRALBP (C and D) (cyan symbols, *n* = 17 eyes for post-injection day 1, *n* = 14 eyes for post-injection days 3–4). The data for wild-type and *Rpe65*^−/−^ controls are the same in (A and C) for ERG a-wave and in (B and D) for ERG b-wave. Error bars for some points in (A–D) are smaller than the symbol size.

**Figure 5:**
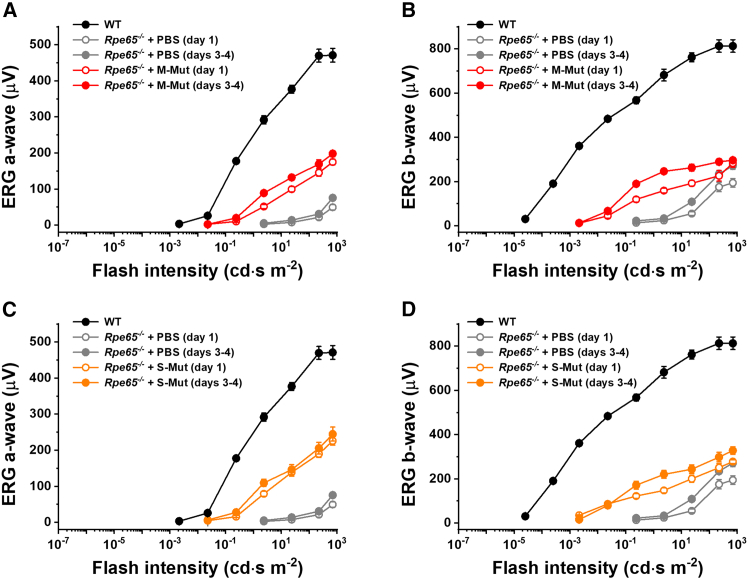
Recovery of scotopic visual function in *Rpe65*^−/−^ mice treated with A212C:T250C mutant CRALBP/9-*cis*-retinal complexes The rod function was studied by *in vivo* ERG. Data show averaged scotopic intensity-response functions (mean ± SEM) for ERG a-wave (A and C) and b-wave (B and D) recorded 1 day (open symbols) and 3–4 days (closed symbols) post-injection in wild-type control (black; untreated, *n* = 8 eyes) and *Rpe65*^−/−^ control (gray; PBS injected, *n* = 9 eyes for post-injection day 1, *n* = 4 eyes for post-injection days 3–4) mice, as well as in *Rpe65*^−/−^ animals injected with mutant monomeric CRALBP (A and B) (red symbols, *n* = 6 eyes for both post-injection times) or with SHMW mutant CRALBP (C and D) (orange symbols, *n* = 6 eyes for both post-injection times). The data for wild-type and *Rpe65*^−/−^ controls are the same in (A and C) for ERG a-wave and in (B and D) for ERG b-wave (reproduced from Figure 4). Error bars for some points in (A–D) are smaller than the symbol size.

ERG recordings confirmed that rod photoreceptors in control PBS-treated *Rpe65*^−/−^ mouse eyes responded to bright light *in vivo*, even in the absence of chromophore. They generated typical ERG waveforms with the initial negative component (a-wave) followed by larger positive ERG b-wave driven by rod ON bipolar cells. As in the case of transretinal recordings (Figure 2), both a- and b-wave ERG components in these animals were significantly lower than those in wild-type mice with normally operating RPE visual cycle and their photosensitivity was reduced dramatically as well (Figure 4). Notably, we found that the intravitreal administration of 9-*cis*-retinal-loaded wild-type, monomeric CRALBP increased maximal response amplitudes and photosensitivities of both ERG a- and b-waves to a significant degree (Figures 4A and 4B). A similar recovery of rod responses was observed in the eyes injected with wild-type SHMW CRALBP (Figures 4C and 4D). In both cases, slightly better ERGs were obtained 3–4 days post-injection in darkness compared to those recorded after 25–30 h (day 1). Importantly, intraocular treatment with both monomeric and SHMW variants of photostable A212C:T250C mutant CRALBP proteins produced comparable recovery of rod function in *Rpe65*^−/−^ mice (Figures 5A–5D). Thus, all four types of CRALBP forms assessed here can supply 9-*cis*-retinal chromophore to rods in intact mouse eyes and promote robust rod pigment regeneration under dark-adapted conditions.

### Accelerated rod dark adaptation in mice treated with CRALBP proteins

Finally, we determined whether the amount of 9-*cis*-retinal chromophore delivered to retinas of *Rpe65*^−/−^ mice via intravitreal injections of either wild-type or A212C:T250C mutant CRALBP variants was sufficient to drive visual pigment regeneration and promote dark adaptation of rods *in vivo*. Normally, the dark adaptation of rods in the intact vertebrate eye is set by the regeneration of their photobleached pigment with fresh 11-*cis*-retinal supplied by the RPE.

**Figure 6:**
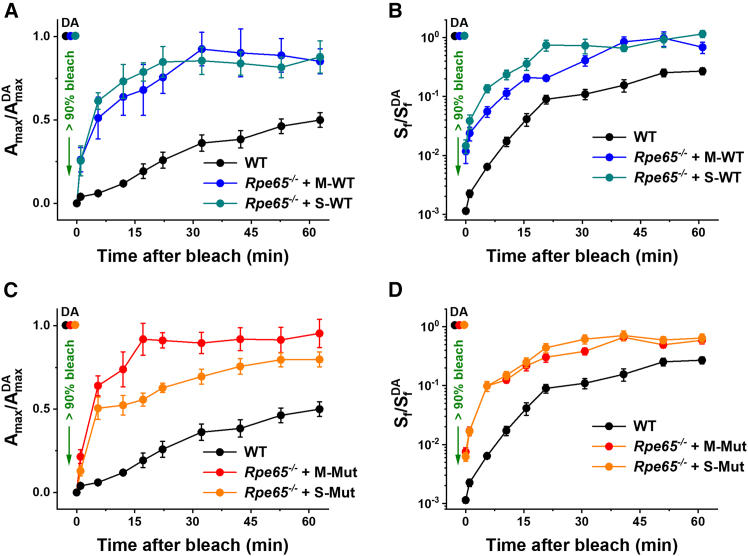
Accelerated dark adaptation of *Rpe65*^−/−^ mice treated with wild-type or A212C:T250C mutant CRALBP proteins loaded with 9-*cis*-retinal The experiments were performed 3–4 days post-injection. (A and B) Recovery of normalized *A*_max_ (A) and flash sensitivity, *S*_f_, (B) after bleaching >90% rhodopsin at time 0 in wild-type control (black; untreated, *n* = 8 eyes) mice, as well as in *Rpe65*^−/−^ animals injected with wild-type monomeric CRALBP (blue symbols, *n* = 4 eyes) or with wild-type SHMW CRALBP (cyan symbols, *n* = 11 eyes) proteins. (C and D) Recovery of normalized *A*_max_ (C) and *S*_f_ (D) after bleaching >90% rhodopsin at time 0 in wild-type control (black; untreated, *n* = 8 eyes) mice, as well as in *Rpe65*^−/−^ animals injected with A212C:T250C mutant monomeric CRALBP (red symbols, *n* = 7 eyes) or with mutant SHMW CRALBP (orange symbols, *n* = 8 eyes) proteins. DA refers to *A*_max_ or *S*_f_ in the dark. Values are means ± SEM. Error bars for some points in (A–D) are smaller than the symbol size.

After recording dark-adapted scotopic ERG responses of RPE65-deficient mice 3–4 days post-injection with CRALBP, their eyes were briefly exposed to bright green light to bleach the bulk (>90%) of their rod pigment, and the recovery of both maximal ERG a-wave amplitude (*A*_max_) and dim flash sensitivity (*S*_f_) was followed in the dark (Figure 6). Immediately after a nearly complete pigment bleach, rods in animals treated with either monomeric or SHMW forms of wild-type CRALBP proteins loaded with 9-*cis*-retinal produced barely detectable a-wave responses that were desensitized by ∼2 log units compared to those in the dark (Figures 6A and 6B). Rod photoresponses then recovered gradually over the following 60 min of dark adaptation, with similar kinetics in animals treated with monomeric or SHMW CRALBP variants. Strikingly, after both treatments, the rod dark adaptation in *Rpe65*^−/−^ mice was significantly faster than that typically observed in wild-type (C56Bl/6) animals (Figures 6A and 6B, black symbols). Similar robust and unusually rapid dark adaptation of rods was also observed 3 days following treatments with both monomeric and SHMW A212C:T250C mutant CRALBP proteins (Figures 6C and 6D). These results demonstrate the direct and efficient delivery of retinoids to free opsin in rods by all four types of loaded CRALBP preparations for efficient pigment regeneration *in vivo*, bypassing the classic RPE visual cycle.

In summary, our findings demonstrate the potential of our approach for treating retinal disorders linked to retinoid deficiency with chromophore-loaded CRALBP proteins.

## Discussion

The present study demonstrates the feasibility of chromophore-loaded CRALBP proteins, both wild-type and a redox-sensitive A212C:T250C mutant, for functional delivery of 9-*cis*-retinal through intravitreal injection to rod photoreceptors in chromophore-deficient mouse models. Multiple previous studies have established the beneficial effect of chromophore delivery in chromophore-deficient animals and patients. Thus, oral administration of 9-*cis*-retinal produces substantial and long-lasting preservation of visual function in dark-reared chromophore-deficient mice.^29^^,^^30^ A 9-*cis*-retinal-loaded combination of poly(D,L-lactide-co-glycolide) microparticles and alginate demonstrated sustained drug release and restored photoreceptor function in mice following subcutaneous injection.^31^ In addition, oral treatment with the chromophore analog 9-*cis*-retinyl acetate could restore light sensitivity of *Rpe65*^−/−^ mice^32^ and, following long-term monthly administration, also counteracted the age-driven deterioration of photoreceptor function in wild-type mice.^33^ Follow-up clinical studies demonstrated improvement of visual function following oral administration of 9-*cis*-retinyl acetate in retinitis pigmentosa patients^34^ and in children with Leber congenital amaurosis and RPE65 and LRAT mutations.^35^ Similarly, conjugated chitosan-9-*cis*-retinal is slowly absorbed from the gastrointestinal tract, resulting in sustainable plasma levels of 9-*cis*-retinol and recovery of visual function in chromophore-deficient mice.^36^

Our findings highlight several novel key aspects potentially relevant for the development of protein-based therapies aimed at treating retinal diseases linked to visual chromophore deficiency. A major innovation of this work lies in the design and biophysical characterization of the di-cysteine CRALBP mutant. Structural modeling suggests that the engineered cysteine residues, located at solvent-accessible and spatially adjacent positions within helices 10 and 12 of CRALBP’s mobile gate, can form a reversible disulfide bond upon oxidation. Indeed, the oxidized form of the A212C:T250C mutant dramatically (by up to 4.3-fold) increased the photostability of bound 9-*cis*-retinal compared to its reduced counterpart. This finding suggests that redox-sensitive locking of the mobile gate conformation could stabilize the ligand-protein complex by preventing photoisomerization and premature release during transit through extracellular environments. Such a mechanism could be particularly beneficial for intravitreal applications, where the stability of the chromophore-loaded protein under physiological light exposure is critical. Future work will seek to demonstrate directly the hypothesized superiority of the redox-sensitive CRALBP over its wild-type counterpart. In addition, the possibility of eliciting an immune or allergic response in the eye by the introduction of engineered redox-sensitive disulfide bonds will have to be investigated and ruled out before therapeutic use.

Our results further show that both wild-type and A212C:T250C CRALBP variants, when loaded with 9-*cis*-retinal, successfully deliver chromophore to RPE65-deficient mouse retinas. This was demonstrated both *ex vivo* using transretinal ERG and *in vivo* via intravitreal injection followed by full-field ERG analysis. Notably, both the monomeric and SHMW forms of each protein restored rod function with comparable efficacy. These data suggest that the redox modulation does not impair the ability of CRALBP to deliver its ligand from the vitreous into the retina and that the engineered disulfide bond does not interfere with its ability to regenerate the visual pigment.

Interestingly, the SHMW fractions, although heterogeneous and composed of higher-order aggregates, were particularly effective in delivering chromophore to photoreceptors *in vivo*, as indicated by IHC and functional analysis. This may reflect a longer retention time or enhanced uptake and transport properties of aggregated CRALBP complexes, a feature worth further exploration in future delivery platform designs. Previous studies on retinal transduction have identified the inner limiting membrane as a major impediment to the intravitreal delivery of nanoparticles.^37^ Our IHC data suggest that chromophore-loaded SHMW CRALBP is capable of penetrating the retina quite well. While our current findings in the rodent model indicate a promising safety profile, translating this approach to larger animals or non-human primates may present challenges, owing to interspecies differences in ILM thickness and retinal architecture, as demonstrated previously.^38^^,^^39^

In both experimental paradigms, isolated retina and whole eye (*in vivo*), the administered CRALBP/9-*cis*-retinal complexes restored visual sensitivity to levels approaching those achieved with free 9-*cis*-retinal, while potentially offering improved stability and bioavailability. Furthermore, dark adaptation kinetics in treated *Rpe65*^−/−^ mice were significantly faster than those observed in wild-type animals, indicating that the chromophore was rapidly accessible to opsin for pigment regeneration. This striking observation suggests that chromophore-loaded CRALBP can overcome blockade of the RPE65-dependent visual cycle by directly supplying rods with the necessary *cis*-retinoid for vision. Overall, this insight will be essential for translating CRALBP particles into a mutation-agnostic shunt-like therapy for improving visual outcomes in patients with inherited retinal disorders. The ability to accelerate dark adaptation is particularly relevant for diseases associated with delayed or incomplete pigment regeneration. Moreover, the use of CRALBP as a carrier could reduce the cytotoxicity and bioavailability issues commonly associated with systemic retinoid administration.

Future studies should investigate long-term safety and efficacy, dosing regimens, and delivery optimization in larger animal models. Additionally, the capacity of CRALBP to deliver visual retinoids to support cone photoreceptor function, which remains an unmet need in patients with central vision loss, should be further examined. Notably, oral gavage of 9-*cis*-retinal has been shown to improve cone function in a mouse model of fundus albipunctatus with disrupted 11-*cis*-retinol dehydrogenase genes.^40^ Combination strategies that leverage CRALBP’s photoprotective properties with gene therapies or sustained-release formulations may also yield synergistic therapeutic effects.

## Materials and methods

### Production of native CRALBP and its A212C:T250C double mutant

Cloning of *Rlbp1* gene and expression of CRALBP protein were carried out essentially as described earlier.^13^^,^^23^ In brief, human *RLBP1* cDNA (JRAUp969D1020D, SEQ ID NO:1) was obtained from the German Center for Genomic Research GmbH and cloned into the NdeI and XhoI sites of the pET-28a(+) vector following the Promega protocol Streamlined Restriction Digestion, Dephosphorylation and Ligation leading to the pET-28a(+) CRALBP overexpression plasmid. BL21(DE3) strains of *E. coli* (Invitrogen) were transformed with the pET-28a(+) CRALBP overexpression plasmid. After inoculating, cells were grown at 20°C in LB medium containing 30 mg/mL kanamycin, induced with 1 mM isopropyl-thiogalacto-pyranoside at an OD(600) of 0.7, harvested at 5,000 × *g* for 45 min, resuspended in ice-cold lysis buffer, and disrupted in a Maximator. The lysate comprising native CRALPB was centrifuged at 20,000 × *g* for 35 min to remove debris.

A212C and T250C point mutations were introduced sequentially by site-directed mutagenesis into the pET-28a(+) CRALBP overexpression using the plasmid primer sequences CATGCAGCAGTGTGCTAGCCTCCGGACTTCAGATCTCAGG (forward for A212C), GGAGGCTAGCACACTGCTGCATGGTAAAGCCCTTGAAGTTC (reverse for A212C), CTTCACCACGTGCTACAATGTGGTCAAGCCCTTCTTGAAG (forward for T250C), and CCACATTGTAGCACGTGGTGAAGTACCATGGCTGGTGGATG (reverse for T250C) according to the instructions described in the QuikChange kit from Stratagene. The presence of the A212C:T250C point mutations in the plasmid was verified by sequencing (Microsynth AG, Balgach). BL21(DE3) cells transformed with A212C:T250C CRALBP mutant overexpression plasmid were cultured and harvested as described for native CRALBP.

Lysates were separately purified by affinity chromatography on Ni-NTA Superflow (QIAGEN) according to the manufacturer’s instructions. Briefly, the lysates were loaded on the column previously equilibrated in a lysis buffer (20 mM Tris-HCl, 100 mM NaCl, pH 7.4) washed with a lysis buffer, and eluted with an elution buffer (20 mM Tris-HCl, 100 mM NaCl, 500 mM imidazole, pH 7.4). Purity and yields were determined by SDS-PAGE and the colorimetric bicinchoninic acid assay (Pierce Chemical Company), respectively.

### Ligand loading and gel permeation chromatography

Freshly prepared apo-CRALBP was concentrated to 15–30 mg/mL using Vivaspin 15R Hydrosart (Sartorius) and dialyzed against 2x PBS to remove imidazole before adding *cis*-retinoids. All procedures involving *cis*-retinals were carried out under dim red illumination (40-W ruby bulbs) at 4°C, unless specified.

Native CRALBP and the di-cysteine A212C:T250C mutant of CRALBP were both loaded with ligands adding aliquots of 9-*cis*-retinal (40 mM) dissolved in pure ethanol at a 1.5 molar excess of the protein solutions and a final ethanol concentration of 1%–2% v/v. The samples were incubated for 30 min at 2°C–4°C and then centrifuged at 15,000 × *g* for 5 min. Unbound retinoids were removed from the ligand complexes through GPC.

Preparative GPC of the native CRALBP *cis*-retinal complex was carried out on a Superdex 200 26/60 (Cytiva, USA) column using GPC buffer (10 mM HEPES, 100 mM NaCl, pH 7.5). Mutant CRALBP was purified on the same column in the presence of GSSG using modified GPC buffer (10 mM HEPES, 100 mM NaCl, 5 mM GSSG, pH 7.5). Both complexes were fractionated at 5-mL intervals. Peak fractions from preparative GPC were pooled, concentrated to approximately 4 mg/mL, and re-chromatographed by analytical GPC using a Superose 6 Increase 10/300 GL column.

### Average size distribution through DLS

Peak fractions from analytical GPC were analyzed via DLS using a Malvern Zetasizer S. Each DLS experiment was conducted according to the parameters outlined elsewhere.^23^ The run duration and number of runs per measurement were optimized using the Malvern Zetasizer S software.

### Photoisomerization assay

Photoisomerization assays of *cis*-retinal and its complexes with wild-type CRALBP or the di-cysteine A212C:T250C mutant of CRALBP were conducted as described previously.^7^ Purified ligand complexes obtained through GPC were diluted to a concentration of 26 μM in GPC buffer (10 mM HEPES, 100 mM NaCl, pH 7.5), with equimolar amounts of BSA added to prevent protein precipitation induced by light-induced free all-*trans*-retinal formation. The samples were exposed to a 100-W daylight bulb, providing an illuminance of 1,000 lux (measured using a Voltcraft MS-1300 Luxmeter), at room temperature in a darkroom setting. UV-vis absorption spectra were recorded at half-minute intervals over a total duration of 20 min using the Evolution diode array UV-vis spectrophotometer (Thermo Scientific). For the preparation of the oxidized A212C:T250C mutant, 5 mM GSSG was introduced to the buffer during preparative GPC of the respective complex. To restore the reduced state, the GSSG was removed by concentrating the samples 3-fold using a Vivaspin 15R Hydrosart (Sartorius) and subsequently re-diluting them in buffer (10 mM HEPES, 100 mM NaCl, pH 7.5) to the original volume. The washed samples were then reduced by adding 5 mM DTT and kept at 4°C for 1 h.

### Animals

Mice with a conventional knockout of the retinal pigmented epithelium protein 65-kDa gene (*Rpe65*^−/−^) were described previously.^41^ Young adult animals of either sex (6–10 weeks old) were used for all experiments. CRALBP-deficient (*Rlbp1*^−/−^) mice used for IHC were also described earlier.^17^ Animals were provided with standard chow (LabDiet 5053; LabDiet, Purina Mills) and maintained under a 12-h light/12-h dark cycle. Mice were dark-adapted overnight before physiological recordings. All experimental protocols followed the Guide for the Care and Use of Laboratory Animals and were approved by the University of California Irvine Institutional Animal Care and Use Committee (protocol #AUP-24-052).

### Acute application of CRALBP proteins loaded with 9-*cis*-retinal to mouse retinas

200-μL aliquots of concentrated (∼55–65 mg/mL) wild-type or A212C:T250C mutant monomeric or SHMW CRALBP proteins (with attached His-tag peptide) in 100 mM NaCl in 10 mM HEPES solution (pH 7.5) containing ∼1.62 mM of 9-*cis*-retinal were diluted in 1.8 mL of L15 cell culture solution (13.6 mg/mL, pH 7.4, Sigma-Aldrich) containing 1% BSA and suspended thoroughly in the dark. The final concentration of the retinoid was estimated to be ∼160 μM. A whole isolated *Rpe65*^−/−^ mouse retina on filter paper was incubated in a Petri dish with 2 mL of this solution in oxygenated light-tight container for 4–4.5 h in the dark, at room temperature. For the control experiments, untreated retinas from the same mouse line were incubated 4–4.5 h in the same L15 solution but without the CRALBP/retinoid complexes. In a separate control experiment, a comparable amount of pure 9-*cis*-retinal in L15 (∼135 μM, dissolved in 0.1% EtOH) was applied to the retina for 1–1.5 h. The tissue was then transferred to the perfusion chamber for *ex vivo* ERG recordings, as described below.

### *Ex vivo* electroretinographic recordings from isolated mouse retinas

RPE65-deficient mice were dark-adapted overnight and sacrificed by CO_2_ asphyxiation and a whole retina was removed from each mouse eyecup under infrared illumination. The retina was mounted on filter paper with the photoreceptor side up and placed in a perfusion chamber between two electrodes connected to a differential amplifier.^42^ The tissue was perfused with Locke’s solution containing 112.5 mM NaCl, 3.6 mM KCl, 2.4 mM MgCl_2_, 1.2 mM CaCl_2_, 10 mM HEPES, pH 7.4, 20 mM NaHCO_3_, 3 mM Na succinate, 0.5 mM Na glutamate, 0.02 mM EDTA, and 10 mM glucose. This solution was supplemented with 2 mM L-glutamate and 10 μM DL-2-amino-4-phosphonobutyric acid (DL-AP4) to block the postsynaptic components of the photoresponse^43^ and with 20 μM BaCl_2_ to suppress the slow glial PIII component.^44^ MEM vitamins and MEM amino acid solutions (Sigma) were also added to improve retina viability. The perfusion solution was continuously bubbled with a 95% O_2_/5% CO_2_ mixture and heated to 36°C–37°C.

Light stimulation was applied in 20-ms test flashes of calibrated 505-nm light-emitting diode (LED) light. The stimulating light intensity was controlled by a computer in 0.5 log unit steps. Intensity-response relationships were fitted with Naka-Rushton hyperbolic functions, as follows:

(Equation 1) $$R=\frac{{R_{\max}\;\cdot \;I}^{n}}{I^{n}+I_{1/2}^{n}}\text{,}$$

where *R* is the transient-peak amplitude of the response, *R*_*max*_ is the maximal response amplitude, *I* is the flash intensity, *n* is the Hill coefficient (exponent), and *I*_1/2_ is the half-saturating light intensity. Photoresponses were amplified by a differential amplifier (DP-311, Warner Instruments), low-pass filtered at 30 Hz (8-pole Bessel), digitized at 1 kHz, and stored on a computer for further analysis. Data were analyzed with Clampfit 10.6 and Origin 2023 software.

### Intravitreal injection of CRALBP proteins loaded with 9-*cis*-retinal

With the room lights off, 2 μL of concentrated (∼55–65 mg/mL) and carefully mixed monomeric or SHMW CRALBP proteins (with attached His-tag peptide) in 100 mM NaCl in 10 mM HEPES solution (pH 7.5) containing ∼1.62 mM of 9-*cis*-retinal were injected into the vitreous of the eye of each anesthetized *Rpe65*^−/−^ mouse, and an equal volume of PBS solution was injected into the control eyes (same or different animals were used for control injections). The injections were performed by hand using a Hamilton syringe, under a dissecting microscope and infrared illumination. Before each injection, a small hole was made in the cornea with a 27G x 1/2 needle to facilitate the insertion of the syringe needle. The total amount of injected retinoid was estimated to be ∼3.2 nmol, which exceeds the average rhodopsin content in the adult wild-type mouse eye by a factor of 5–6. Animals were then allowed to recover from the anesthesia and returned to their original cages with access to food and water and kept in the dark for 25–30 h (day 1) prior to recordings of intensity-response families by ERG *in vivo*, as described below. Mice were then recovered in the dark and re-tested after additional 48–72 h (days 3–4).

### *In vivo* ERG recordings

Dark-adapted *Rpe65*^−/−^ mice were anesthetized with an intraperitoneal injection of a mixture of ketamine (100 mg/kg) and xylazine (4 mg/kg). Pupils were dilated with a drop of 1% atropine sulfate. Mouse body temperature was maintained at 37°C with a heating pad. ERG responses were recorded from both eyes by corneal contact electrodes held in place by a drop of Gonak solution. Full-field ERGs were performed with the UTAS BigShot apparatus (LKC Technologies) using Ganzfeld-derived test flashes of calibrated green 530-nm LED light (within a range from 2.2 × 10^−4^ cd⋅s m^−2^ to 23.5 cd⋅s m^−2^) or white light generated by the Xenon Flash tube (from 80.7 cd⋅s m^−2^ to 700 cd⋅s m^−2^).^45^ Both scotopic ERG a-waves and b-waves were measured on days 1 and 3–4 after injection of CRALBP samples.

For rod dark adaptation tests (performed on days 3–4 after injections), rod ERG a-wave flash sensitivity (*S*_f_) was first determined in the dark, as follows:

(Equation 2) $$S_{f}=A/\left(A_{\max}\times I\right)\text{,}$$

where *A* is the rod a-wave dim flash response amplitude, *A*_*max*_ is the maximal response amplitude for that eye produced with the brightest green light stimulus (23.5 cd⋅s/m^2^), and *I* is the flash strength of dim flash response (in cd⋅s m^−2^). The rod pigment was then bleached nearly completely by a 35-s exposure to bright light delivered by a 520-nm LED focused at the surface of the mouse eye cornea. The bleaching fraction was estimated by the following formula:

(Equation 3) F=1–exp(–I×P×t),

where *F* is the fraction of pigment bleached, *t* is the duration of the light exposure (in seconds), *I* is the bleaching light intensity of 520-nm LED light (1.3 × 10^8^ photons μm^−2^ s^−1^), and *P* is the photosensitivity of mouse rods at the wavelength of peak absorbance (5.7 × 10^−9^ μm^2^).^46^ After the bleach, the recovery of the rod responses was followed in darkness for up to 1 h. Mice were re-anesthetized with a lower dose of ketamine (∼1/3 of the initial dose) in the middle of that period. If necessary, a small drop of PBS solution was gently applied to the eyes with a plastic syringe to protect them from drying and maintain contacts with the recording electrodes.

### Immunohistochemistry

Mice were injected with CRALBP proteins loaded with retinoids and euthanized 36–48 h post-injection, as described above, and their eyes were enucleated and placed in 1x PBS. A puncture was made at the limbus using a 23G needle, and the eye was then placed in room temperature in 1x PBS containing 4% (w/v) paraformaldehyde (Sigma-Aldrich) to fix the tissue. The eye was placed back into 1x PBS, and the anterior segment was removed by dissection. The resulting eyecup was fixed for an additional 15–30 min at room temperature. After fixation, the eyecups were sequentially incubated at room temperature in 1x PBS containing 5%, 10%, and 20% (w/v) sucrose (MilliporeSigma) for 30 min at each concentration. The eyecups were then transferred into a 1:1 mixture of 30% sucrose and O.C.T. compound (Fisher Scientific) and incubated overnight at 4°C. The next day, the eyes were embedded in the same 30% sucrose/O.C.T. mixture and frozen on top of a dry ice-chilled metal block. Retinal sections were cut at a thickness of 10 μm at −20°C using a Leica CM1850 cryostat and stored at −80°C until needed. They were rehydrated with 1x PBS and then blocked and permeabilized for 30 min with 0.2% (v/v) Triton X-100 (Millipore Sigma) and 5% normal goat serum (Abcam) in 1x PBS. The sections were then incubated overnight with anti-CRALBP primary antibody (1:1,000; J. Saari, UW55) in a solution containing 0.2% Triton X-100 and 5% normal goat serum in 1x PBS. The sections were washed with 1x PBS 3 times and then incubated with Alexa Fluor 594-conjugated goat anti-rabbit IgG (1:250; Thermo Fisher Scientific, A11037) in 1x PBS. They were then washed with 1x PBS 5 times and mounted with VECTASHIELD Mounting Medium containing DAPI (Vector Laboratories) for imaging. Fluorescence and bright-field images were acquired with a Keyence BZ-X800 All-in-One fluorescence microscope.

### Statistics

All biochemical and electrophysiological data were expressed as means ± SEM and analyzed using the independent two-tailed Student’s *t* test, with an accepted significance level of *p* < 0.05.

## Data and code availability

All data generated and analyzed during this study will be provided upon reasonable request

## Acknowledgments

This work was supported by NanoRetinal Inc., 10.13039/100000002NIH grants EY035137 (V.J.K.) and EY034519 (P.D.K.), and 10.13039/100000738Department of Veterans Affairs grants I02BX004939 and IK6BX006800 (P.D.K.). We are grateful for the support from the Department of Chemistry, Biochemistry and Pharmaceutical Sciences, University of Bern. The authors acknowledge support to the Gavin Herbert Eye Institute at the University of California, Irvine, from an unrestricted grant from Research to Prevent Blindness and from 10.13039/100000002NIH core grant P30 EY034070. We are grateful to Dr. Krzysztof Palczewski for his comments on an earlier version of the manuscript.

## Author contributions

J.C., A.S., and V.J.K. conceived the study and directed the research. A.V.K., W.A., M.H., and P.D.K. designed and conducted experiments and analyzed data. A.V.K., W.A., A.S., and V.J.K. wrote the manuscript. J.C., P.D.K., A.S., and V.J.K. obtained funding and provided essential resources. All authors read and approved the final version of the manuscript.

## Declaration of interests

A.S., J.C., and W.A. are shareholders and received honoraria and research contracts from NanoRetinal Inc. involved in development of therapies for retinitis pigmentosa. A.S. and W.A. are inventors on the patent WO 2024/110625 A1 related to human therapies for retinitis pigmentosa.

## Supplemental information

Document S1. Figures S1–S4

Document S2. Article plus supplemental information
