# Supplementary material for: Chromophore-loaded CRALBP mutant proteins restore rod function in chromophore-deficient mice
Source: Mol Ther Adv. 2026 Feb 17;34(1):201701. doi: 10.1016/j.omta.2026.201701 (PMC13148898; doi:10.1016/j.omta.2026.201701)
Supplement: Document S1. Figures S1–S4 [file mmc1.pdf]

OMTA, Volume *34*

Supplemental information

Chromophore-loaded CRALBP mutant proteins restore rod function in chromophore-deficient mice

Alexander V. Kolesnikov, Walter Aeschimann, John Cullity, Maximilian Halabi, Philip D. Kiser, Achim Stocker, and Vladimir J. Kefalov

SUPPLEMENTAL INFORMATION

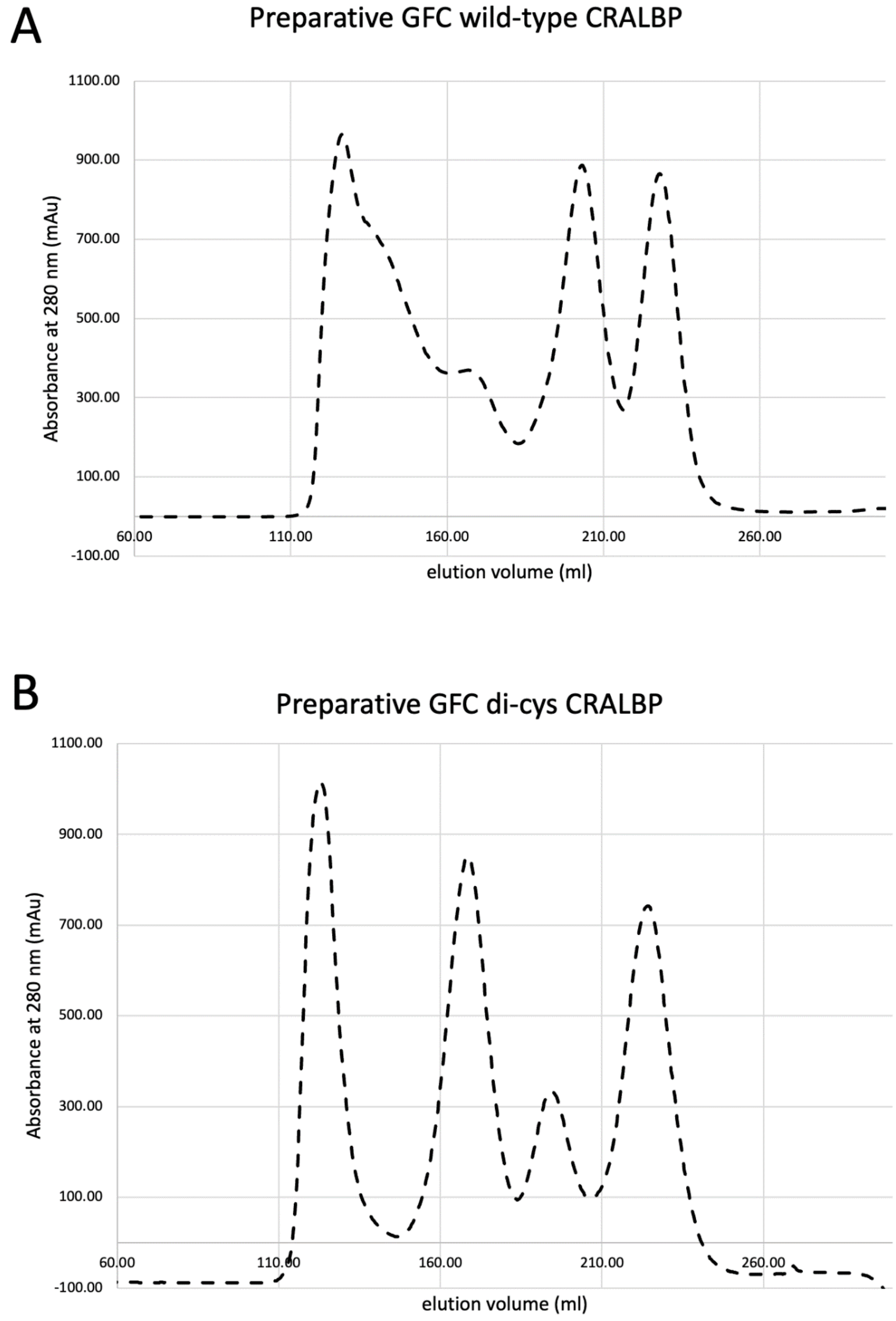

Figure S1. UV/Vis absorption traces of preparative GPC at 280 nm conducted on a Superdex® 200 26/60 column

(A) Native CRALBP after loading with 9-*cis*-retinal and (B) di-cysteine A212C:T250C mutant ligand complex. Specifically, from left to right, the peaks correspond to the super high

molecular weight (SHMW) fraction, high molecular weight (HMW) fraction, dimeric fraction and monomeric fraction, respectively. The figure was adapted from[1].

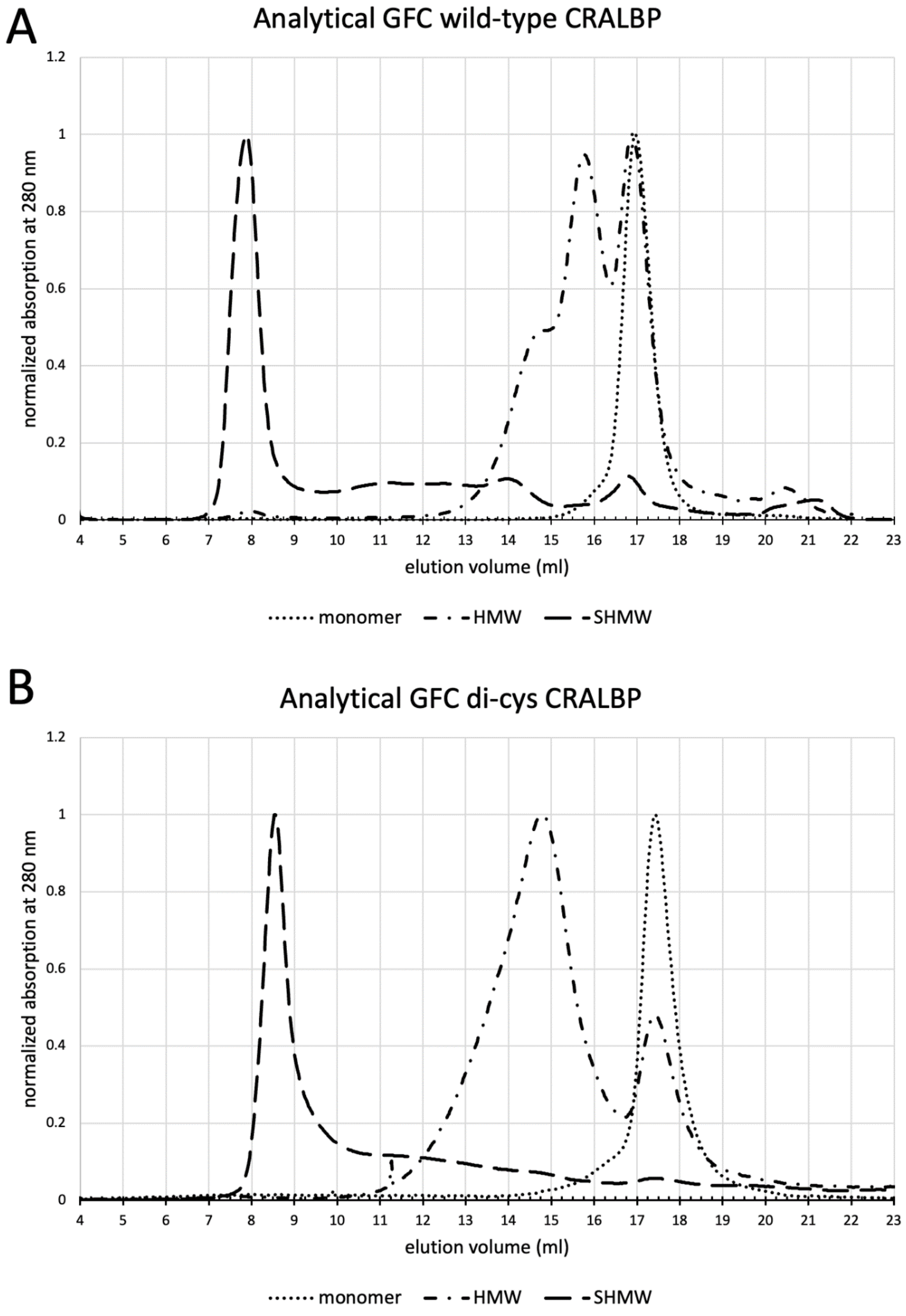

Figure S2. Overlay of UV/VIS absorption traces at 280 nm of analytical GPC runs using a Superose® 6 Increase 10/300 GL column

The SHMW, HMW and monomeric CRALBP peak fractions from preparative GPC were pooled and concentrated to 4mg/ml. The figure was adapted from[1].

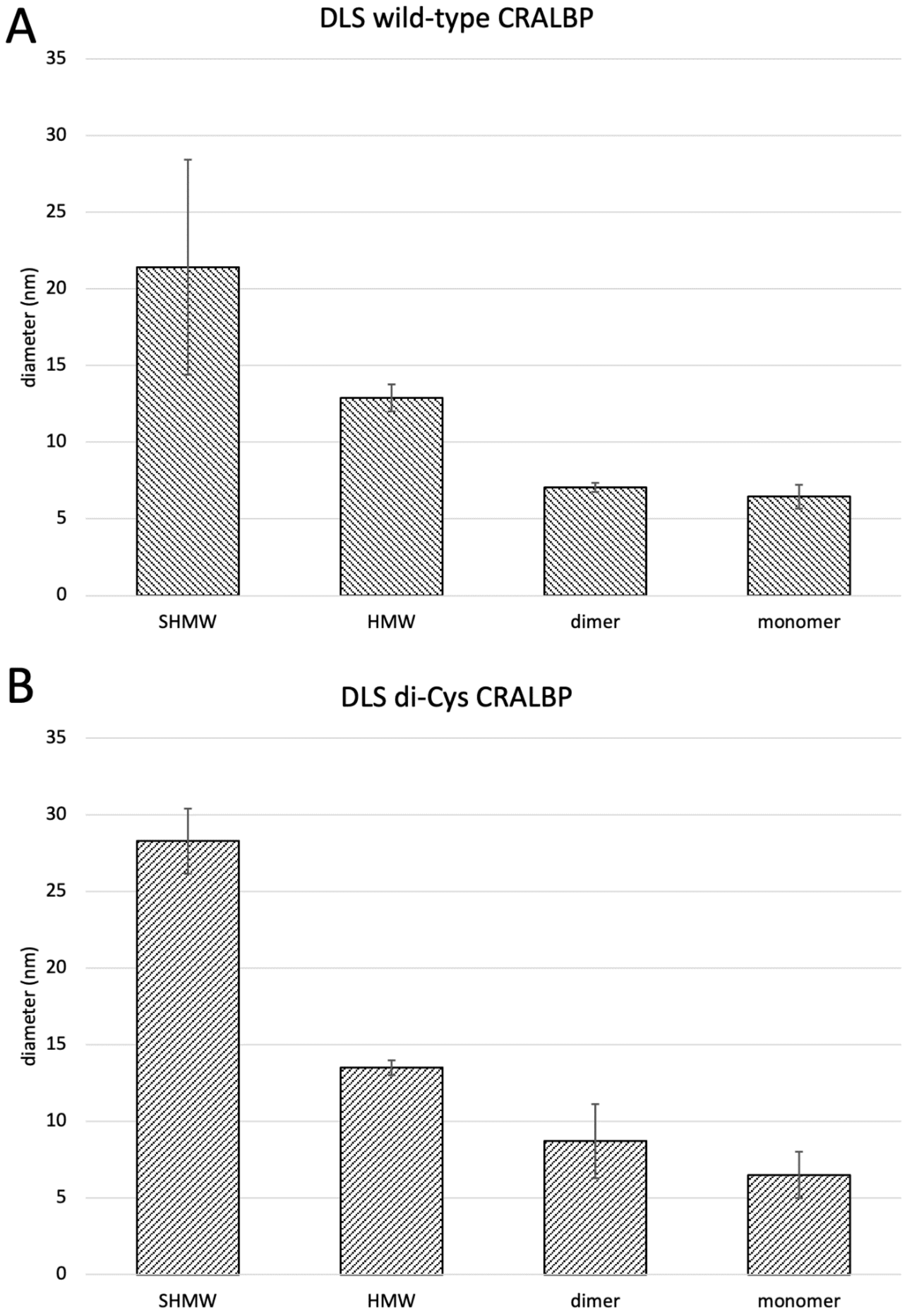

Figure S3. Size distribution of CRALBP ligand complexes determined by DLS on a Malvern Zetasizer S®

Bar chart shows the average size, including error (± SD), of different complex fractions of native CRALBP and CRALBP di-cysteine A212C:T250C mutant after loading with 9-*cis*-retinal. (A) Complex of monomeric CRALBP with 9-*cis*-retinal (6.4 ± 0.8 nm), dimeric complex (7.1 ± 0.3 nm), HMW (12.9 ± 0.9 nm), and SHMW (21.4 ± 7.1 nm), respectively. (B) Di-cysteine A212C:T250C mutant: monomeric CRALBP with 9-*cis*-retinal (6.5 ± 1.5 nm), dimeric complex (8.7 ± 2.4 nm), HMW (13.5 ± 0.5 nm), and SHMW (28.3 ± 2.1 nm), respectively. The figure was adapted from[1].

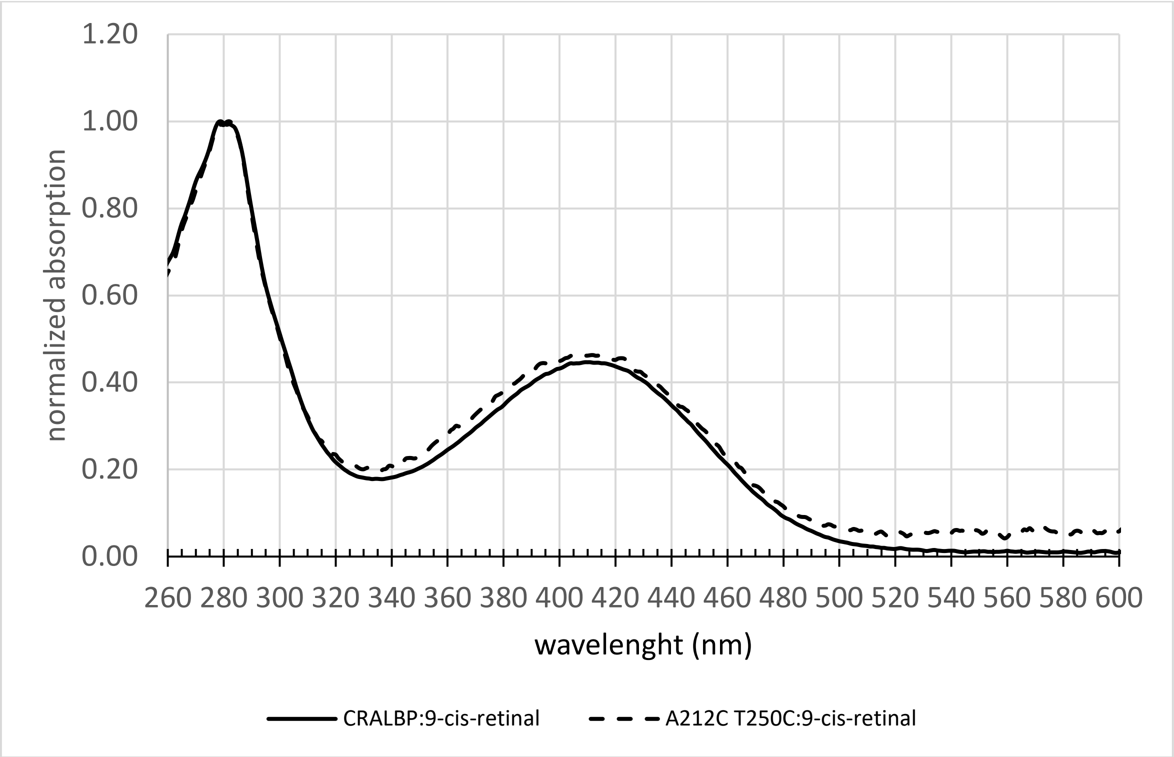

Figure S4. UV-Vis absorption spectra of monomeric native CRALBP and the A212C:T250C mutant in complex with 9-*cis*-retinal

Spectra were normalized to the maximum absorption of the protein at approximately 280 nm. Notably, the absorption maximum for 9-*cis*-retinal bound to CRALBP occurs at 400 nm.

Measured ε280/ε400 ratios for native CRALBP:9-*cis*-retinal and for the A212C:T250C mutant:9-*cis*-retinal were 2.3 and 2.2, respectively. The figure was adapted from[1].

SUPPLEMENTAL REFERENCES

1. Stocker, A. (2022). Redox sensitive Cralbp mutant proteins (patent). https://lens.org/107-851-583-721-884. Univ. Bern.
